# Syndrome cave supérieur secondaire à la pose d'un cathéter veineux central chez un nouveau-né

**DOI:** 10.11604/pamj.2015.20.305.6118

**Published:** 2015-03-30

**Authors:** Mohamed Zouari, Mahdi Ben Dhaou

**Affiliations:** 1 Service de Chirurgie Pédiatrique, CHU Hédi Chaker, 3029 Sfax, Tunisie

**Keywords:** Syndrome cave supérieur, cathéter veineux central, un nouveau-né, Superior vena cava syndrome, central venous catheter, newborn

## Image en medicine

**Figure 1:**
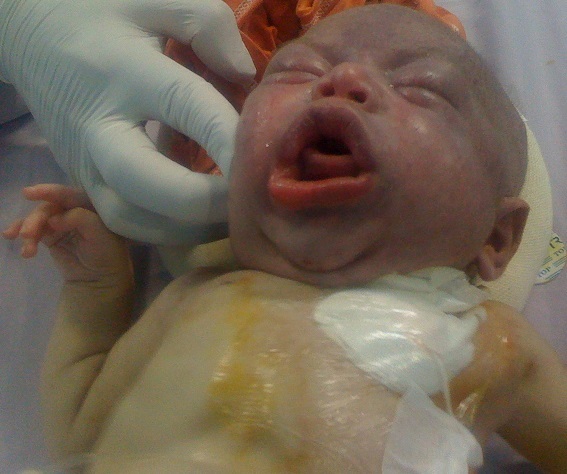
L’œdème facial et la cyanose en rapport avec la ligature des deux veines jugulaires internes chez notre patient

Le recours aux cathéters veineux centraux (CVCs) est fréquent en milieu pédiatrique et néonatal. La mise en place chirurgicale de ces cathéters nécessite le plus souvent la ligature de la veine jugulaire interne (VJI) homolatérale. Nous rapportons le cas d'un nouveau né de sexe féminin, prématuré à 30 semaines d'aménorrhée, admis à l’âge de 50 jours (âge corrigé à 37 semaines d'aménorrhée) pour pyélonéphrite gauche compliquée d'abcès rénaux, arthrite septique multifocale et état de choc septique. La naissance a été faite par césarienne pour une rupture prématurée des membranes de 5 jours. Le poids de naissance a été de 1600 g. Devant la nécessité d'une alimentation parentérale et d'une antibiothérapie adaptée par voie veineuse, un CVC de type Broviac a été mis en place au niveau de la VJI droite. L’évolution a été marquée par l'ablation accidentelle du cathéter au bout de 24 heures. La VJI droite étant sacrifiée, on a opté pour la pose d'un deuxième cathéter de type Broviac au niveau de la VJI gauche. L’évolution a été marquée par l'installation, au bout de 6 heures, d'un œdème de la face et du cou avec dyspnée et cyanose. L'ablation du cathéter avec reprise de la même incision cervicale, identification et anastomose des deux bouts de la VJI gauche a permis de reperméabiliser cette veine avec régression rapide de l’œdème. Figure 1

